# Comparisons of assessment pathways after abnormal mammography screening in Denmark, Norway, and Spain

**DOI:** 10.1007/s10549-023-07219-0

**Published:** 2024-01-29

**Authors:** Susanne Fogh Jørgensen, Silje Sagstad, Javier Louro, Marta Román, Xavier Castells, Solveig Hofvind, Sisse Njor

**Affiliations:** 1 University Research Clinic for Cancer Screening, Randers Regional Hospital, Skovlyvej 15, 8930 Randers NE, Denmark; 2 Section for Breast Screening, Cancer Registry of Norway, Oslo, Norway; 3 Department of Epidemiology and Evaluation, IMIM (Hospital del Mar Medical Research Institute), Barcelona, Spain; 4 Research Network on Chronicity, Primary Care and Health Promotion (RICAPPS), Barcelona, Spain; 5 Department of Health and Care Sciences, The Arctic University of Norway, Tromsö, Norway; 6 Department of Clinical Medicine, Aarhus University, Aarhus, Denmark; 7 Department of Data, Innovation and Research, Lillebaelt Hospital, Beriderbakken 4, 7100 Vejle, Denmark

**Keywords:** Breast neoplasm, Mass screening, Mammography, Sensitivity

## Abstract

**Purpose:**

To ensure high-quality screening programmes and effective utilization of resources, it is important to monitor how cancer detection is affected by different strategies performed at recall assessment. This study aimed to describe procedures performed at recall assessment and compare and evaluate the performance of the assessment in Denmark, Norway, and Spain in terms of screen-detected cancer (SDC) and interval cancer (IC) rates.

**Methods:**

We included women aged 50–69 years from Denmark, Norway, and Spain, who were recalled for assessment after screening mammography, and recorded all procedures performed during six months after diagnosis, and the timing of the procedures. Women were followed for two years and screen-detected and interval cancer, and sensitivity of recall was calculated and compared.

**Results:**

In total, data from 24,645 Danish, 30,050 Norwegian, and 41,809 Spanish women were included in the study. Most of the women had some assessment within 2 months in all three countries. SDC rates were higher in Denmark (0.57) and Norway (0.60) compared to Spain (0.38), as were the IC rates, i.e. 0.25 and 0.18 vs. 0.12, respectively. The sensitivity of the diagnostic follow-up was somewhat higher in Denmark (98.3%) and Norway (98.2%), compared to Spain (95.4%), but when excluding non-invasive assessment pathways, the sensitivities were comparable.

**Conclusion:**

This comparison study showed variation in the assessment procedures used in the three countries as well as the SDC and IC rates and the sensitivity of recall.

## Introduction

Organized population-based mammography screening has been available for decades in several European countries, and participation rates in Western Europe are generally above 75% [1].

Women with abnormal screening examinations are recalled for further assessment [2]. The assessment could include clinical examination of the breast, additional imaging, i.e. extra mammographic images and/or ultrasound, and needle biopsy, which is usually referred to as “triple assessment” [2]. Insufficient assessment, i.e. missing recommended procedures or delays in assessments may affect the prognosis for women with potential breast cancer [3, 4]. Furthermore, delays in the assessment and diagnostic follow-up may cause distress for the women [5].

To ensure high-quality screening programmes and effective utilization of resources it is important to monitor how cancer detection is affected by different strategies performed at recall assessment [2, 6]. Sharing data and information on how screening programmes perform and describing variations between countries might contribute to a discussion on how to improve the effectiveness and feasibility of screening programmes. This study aimed to compare and evaluate the performance of the assessment in Denmark, Norway, and Spain in terms of SDC and IC rates.

## Material and methods

### Study design

This retrospective cohort study included women screened for breast cancer through organized mammography screening programmes in Denmark and Norway during 2016–2019 and in Spain during 2012–2015. Women aged 50–69 years were included in the study group if they had an abnormal screening mammogram recommending further assessment. Only the first recall during the study period was included. We excluded women with a diagnosis of breast cancer before the screening examination, and death or emigration within 1 month, where data were available (Denmark and Norway). The included women were followed through their diagnostic follow-up until a breast cancer diagnosis, or the mammographic findings were ruled out and the women returned to routine screening (max. two years).

### Settings

Following the European guidelines for mammography screening [2] all three countries offer biennial population-based two-view digital mammography screening to women aged 50–69. The screening mammograms are classified into positive or negative based on the assessment consensus of two independent breast radiologists, disagreements assessed by a third radiologist [7].

The Danish programme was gradually implemented in 1991 in one municipality and became nationwide in 2010 [8]. According to Danish legislation, women should have the screening result within 10 working days and an appointment for diagnostic mammography within an additional 6 working days [7, 9]. Recent numbers show coverage of 79% and yearly participation rate of 83% [10].

In Norway, the national screening programme, BreastScreen Norway, started in 1996 as a pilot programme and became nationwide in 2005 [11]. The Cancer Registry of Norway administers the programme. Independent double reading with consensus is standard practice for initial interpretations. All examinations are given a score of 1–5 for each breast and cases with a score of 2 or higher by one or both radiologists are discussed at a consensus meeting where it is decided whether to recall the woman for further assessment, which usually takes place within 1–2 weeks [12]. The annual attendance rate is 75%, and coverage is about 84% [13].

In Spain, the first regional population-based mammography screening programme was launched in Navarre in 1990 and extended to the whole country in 2006. The programme is part of the National Health System strategy [14], however, managed in a decentralized manner structured by geographical regions. Two independent breast radiologists interpret the screening mammogram according to the Breast Imaging Reporting and Data System (BI-RADS) scale [15]. Independent double reading with consensus or arbitration by a third radiologist is standard practice. Those with abnormal mammography defined as BI-RADS scores 3, 4, 5, or 0 are recalled for additional assessments. Further assessment usually takes place within 2 weeks after the screening examination. Data from 2016 indicate that screening coverage in Spain was higher than 90% and that overall participation is 75.7% [16].

### Data sources

The Danish study population was identified using data from the National Breast Cancer Screening database [8]. Diagnostic procedures after screening and cancers were retrieved from the National Cancer Registry [17], The National Pathology Register [18], and the National Patient Registry [19]. Furthermore, information on death and emigration was retrieved from the Civil Registration System in Denmark [20] and different health registries merged using the individual identification number.

In Norway, it has been mandatory to report cancer cases since 1953. Information about assessment procedures and diagnosis among screening participants is stored in databases at the Cancer Registry of Norway. Data about death and emigration were extracted from the Population Registry. Data were merged using the 11-digit personal identification number assigned to all residents.

In Spain, data were obtained from nine different units of the Spanish Breast Cancer Screening Program. All screening examinations performed in these centres between 2012 and 2015 were included. The different centres gather information on screening mammograms, recalls, additional assessments, and diagnoses performed in their defined recruitment areas and they are considered representative of the general Spanish population [21, 22]

### Definitions

The assessment pathways were defined by including all relevant procedures after a positive screening examination and categorized based on the type of assessment and diagnostic procedures performed, i.e. whether additional (mammographic) imaging (including also tomosynthesis and breast MRI), ultrasound examinations or biopsies (core and fine-needle) were performed for a period of 6 months. Women who had no follow-up procedures registered within six months after the screening date were categorized as having ‘No follow-up/lost to follow-up’. The procedures had to be registered before or on the same day as the cancer diagnosis was registered, to be included as recall assessment and not part of pre-operative examinations and cancer treatment.

For Danish and Norwegian women, those cancers diagnosed within six months after the positive screening examination were considered SDC, and those diagnosed from six to 24 months after a positive screening examination or 0–24 months after a negative examination and before the next screening were considered IC. In Spain, all SDC are consistently recorded in the screening programmes databases. The information on interval cancers is actively obtained on a systematic basis using screening centre databases, hospital-based cancer registries, regional Minimum Data Sets, and population-based cancer registries.

SDC’s and IC’s were all defined as invasive carcinomas of the breast and ductal carcinoma in situ using either ICD codes (ICD-10: C50*, D05*).

### Analyses

Distributions of the diagnostic follow-up pathways were presented as absolute numbers with proportions of women referred to the particular assessment and follow-up out of all screened women to account for the varying referral rates in the populations. In Table 5, numbers are presented out of women referred for recall assessment.

Time to first follow-up was calculated in calendar days from the screening examination until the first registered diagnostic follow-up procedure and presented as cumulative proportions, by the final assessment outcome, i.e. false positive or SDC.

The positive predictive value of the diagnostic follow-up (PPV-1) was calculated as the number of SDC’s in each diagnostic pathway divided by the number of women referred to the specific follow-up pathway. In addition, we calculated the positive predictive value of a diagnostic assessment including at least one biopsy, i.e. PPV-2, as a biopsy is needed to have the cancer diagnosis confirmed in most cases. Cancer detection rates were calculated as the number of SDC’s among all screening examinations in the period. IC rates were presented as (1) the number of cancers diagnosed after a negative screening, but before the next screening or 24 months, whatever comes first, per 1000 screening examinations, i.e. IC_rate_, and (2) the number of cancers diagnosed after a false positive screening in each diagnostic follow-up pathway per 1000 false positive recalls, i.e. ICR_pos_.

The sensitivity of screening was defined as the number of SDC’s divided by the total number of cancers (SDC and IC), i.e. Sens-1 [6]. To diminish the potential effect of different referral rates in the populations, we also calculated the sensitivity of the diagnostic pathway by dividing the number of SDC by the IC diagnosed for two years in the referral population, i.e. Sens-2.

## Results

### Study population

**Fig. 1:**
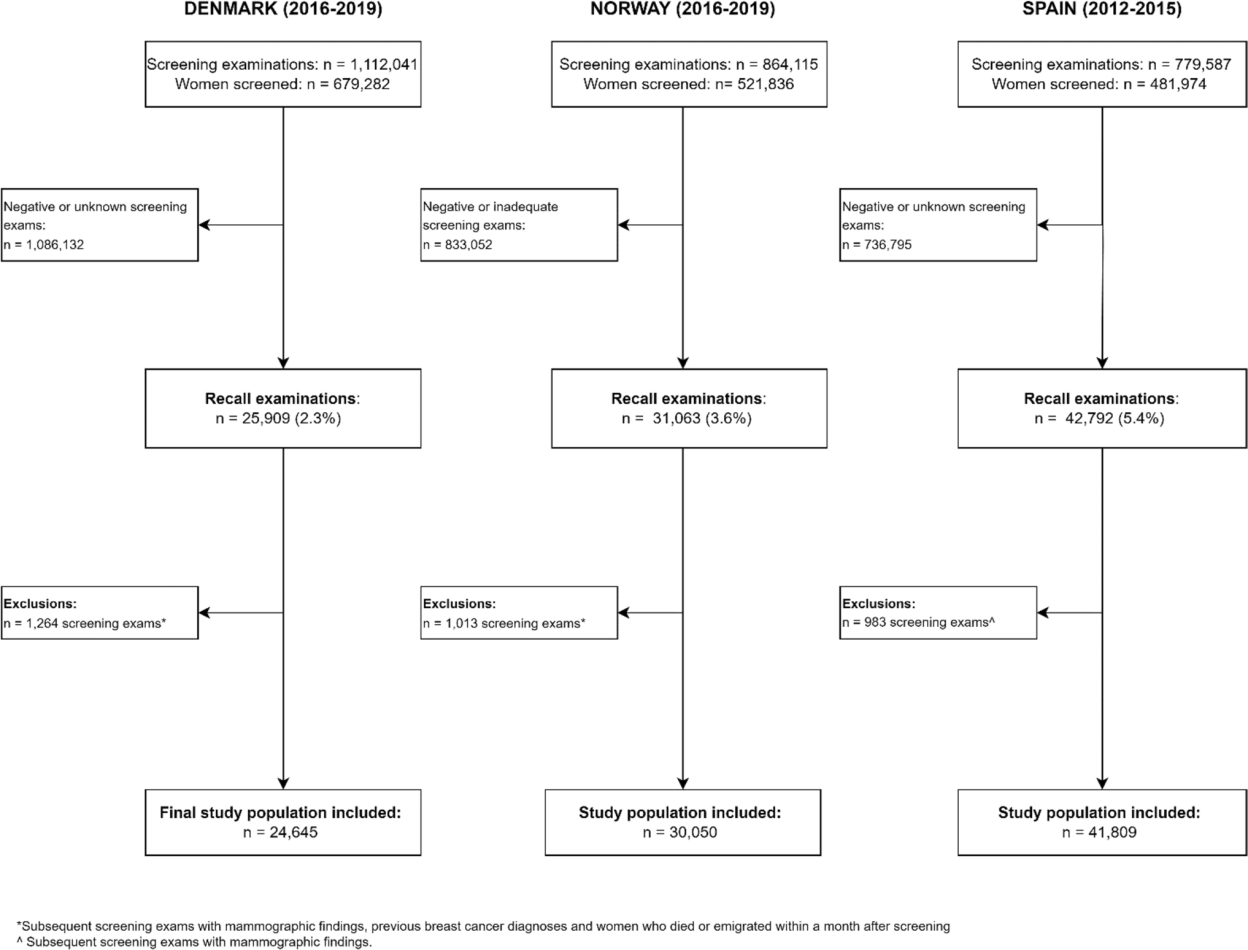
Inclusion and exclusion in the three populations

During the four-year inclusion period, 1,112,041 screening examinations were performed among 679,282 Danish women, 864,115 among 521,836 Norwegian women, and 799,587 among 481,974 Spanish women (Fig. 1). After exclusions, we included 24,645 women with an abnormal screening examination in Denmark, 30,050 from Norway, and 41,809 from Spain. The recall rate was 2.3% in Denmark, 3.6% in Norway, and 5.4% in Spain.

The overall proportion having their first recall assessment within one month after the screening was 85%, in the Danish, 62% in the Norwegian, and 77% in the Spanish population (data not shown).

**Fig. 2:**
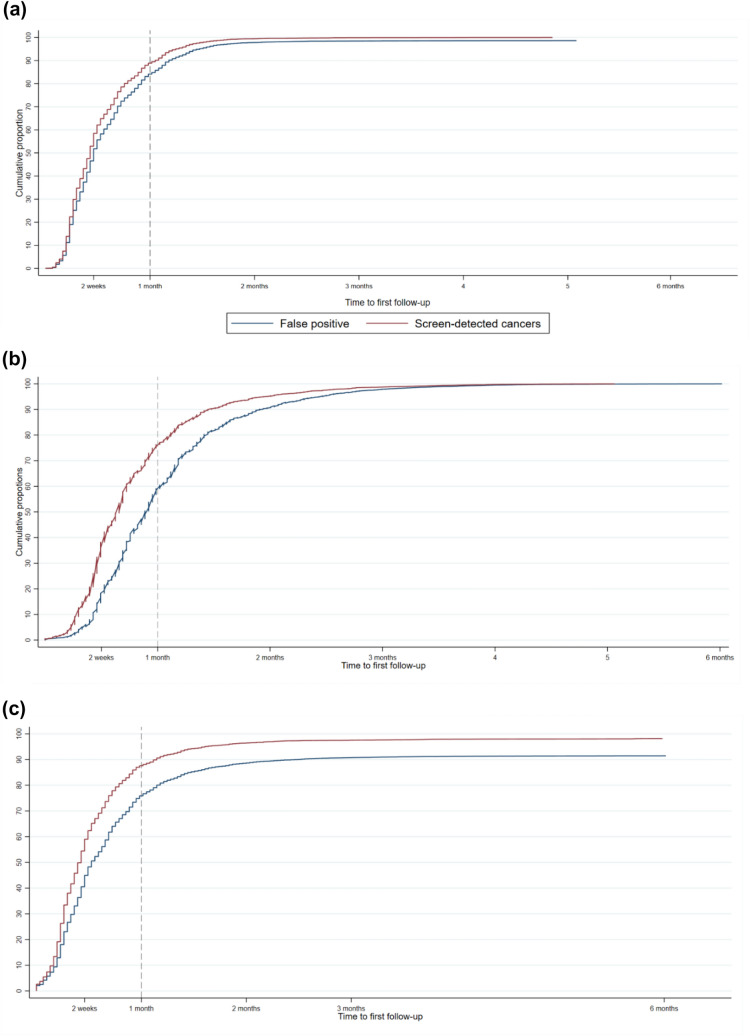
Time to first follow-up by false positive screening mammograms and screen-detected cancers in Denmark (**a**) Norway (**b**) and Spain (**c**)

In all three populations, the proportion who had the first assessment within one month was slightly higher among women with versus without breast cancer diagnosed (Fig. 2). While these differences diminished over time in the Danish and Norwegian populations, they remained stable in the Spanish population, with the lowest follow-up proportions among women categorized as false positive (Fig. 2).

### Overall performance of screening

The overall positive predictive value (PPV-1) of further assessment was 25.7% in Denmark, 17.3% in Norway, and 7.1% in Spain, while corresponding values of PPV-2 of invasive procedures were 58.6%, 40.7%, and 38.8%, respectively (Table 1). The overall rate of SDC in the study period was 0.57% in Denmark (87% invasive), 0.60% in Norway (81% invasive), and 0.38% in Spain (81% invasive), while the rates of IC were 0.25, 0.18, and 0.12 in Denmark, Norway, and Spain, respectively. The IC_pos_ was 0.60 in Denmark (*N* =  ~ 109/ ~ 18,296), 0.37 in Norway (*N* =  ~ 93/ ~ 24,855), and 0.38 in Spain (*N* = 149/ ~ 38,856). The sensitivity of the screening examinations was 70.0% in Denmark, 76.5% in Norway, and 75.9% in Spain, and among the referral women, the sensitivity of the assessment was 98.3% in the Danish referral population, 98.2% in the Norwegian, and 95.4% in Spain (Table 1).

**Table 1:** Overall screening performance indicators by country

	Screening performance indicators		
	Denmark *N* (%)	Norway *N* (%)	Spain *N* (%)
Total number of SDC/All recall examinations (PPV-1)^a^	> 6344/24,645 (25.7)	> 5190/30,050 (17.3)	> 2948/41,809 (7.1)
Total number of SDC among biopsies performed/All biopsies performed (PPV-2)^b^	6336/10,809 (58.6)	5167/12,689 (40.7)	2826/7284 (38.8)
Total number of SDC/All screening examinations (SDC_rate_)^c^	> 6344/ 1,112,041 (0.57)	> 5190/864,115 (0.60)	> 2948/779,587 (0.38)
Total number of interval cancers/All screening examinations without SDC (IC_rate_)^d^	> 2762/1,105,697 (0.25)	> 1589/858,920 (0.18)	934/776,602 (0.12)
Interval cancers/Number of positive screenings not diagnosed with cancer, (IC_pos_)^e^	> 109 / > 18,296 (~ 0.60)	> 93 / > 24,855 (~ 0.37)	> 149/ > 38,856 (0.39)
Number of SDC/Total number of cancers (Sens-1)^f^	> 6344/ > 9106 (70.0)	> 5190/ > 6789 (76.5)	> 2948/3882 (75.9)
Number of SDC/Total number of cancers detected after a recall (Sens-2)^g^	> 6344/ > 6453 (98.3)	> 5190/ > 5283 (98.2)	> 2948/ > 3089 (95.4)

Due to General Data Protection Regulation (GDPR), cells with numbers below 5 and the corresponding denominators (regardless of which tables they occur in) are masked with < / >

*SDC* Screen-detected cancer

^a^Positive predictive value of a positive screening (PPV-1)

^b^Positive predictive value of biopsy (PPV-2)

^c^Cancer detection rate (SDC_rate_)

^d^Interval Cancer rate (IC_rate_)

^e^Interval Cancer rate after positive examination (ICR_pos_)

^f^Sensitivity of screening (Sens-1)

^g^Sensitivity of recall (Sens-2)

### Recall assessment

The referred population could be categorized into eight different recall assessment pathways based on the combinations of the procedures performed (Table 2).

**Table 2:** Diagnostic follow-up pathways during two years from screening and proportions out of all women screened within the study period in Denmark, Norway and Spain

		Denmark (*N* = 679,282 women) *N* (%)	Norway (*N* = 521,836 women) *N* (%)	Spain (*N* = 481,974 women) *N* (%)
1	No follow-up in 6 months./lost to follow-up	248 (0.04)	309 (0.06)	3016 (0.63)
2	Additional imaging^, ultrasound and biopsies	10,446 (1.54)	11,273 (2.16)	1603 (0.33)
3	Additional imaging^ and ultrasound	12,851 (1.89)	14,629 (2.80)	4339 (0.90)
4	Additional imaging^ alone	47 (0.01)	–	7726 (1.60)
5	Ultrasound alone	624 (0.09)	2423 (0.46)	19,093 (3.96)
6	Ultrasound and biopsy	276 (0.04)	1416 (0.27)	3731 (0.77)
7	Additional imaging^ and biopsy	66 (0.01)	–	821 (0.17)
8	Biopsy only (i.e. excisional biopsies, stereotactic biopsies)	87 (0.01)	–	1129 (0.23)
9	Early follow-up recommendation*	–	–	351 (0.07)

^Includes, extra mammography, breast MRIs or tomosynthesis

*Only in Spain

Furthermore, a ninth pathway occurred in the Spanish population, as they referred a minor fraction of the women to an early recall mammogram, in accordance with their guidelines. This was not recommended in Denmark and Norway.

We found 4/10.000 (248/679,282) screened women without follow-up within 6 months in Denmark, 6/10.000 (309/521,836) women in Norway, and 63/10.000 (3016/481,974) screened women in Spain (Table 2).

Among women screened in the study period, 1.54% (95% CI 1.51–1.57%) had at least one biopsy during the follow-up in Denmark, 2.43% (95% CI 2.39–2.47%) in Norway and 1.51% (95% CI 1.48–1.55%) in Spain (Table 2). Furthermore, 3.56% (95% CI 3.52–3.61%) in Denmark were referred for assessment including ultrasound, while the corresponding proportions in Norway and Spain were 5.97% (96% CI 5.90–6.04%) and 5.70% (95% CI 5.64–5.76%), respectively. The proportion who had an ultrasound as the only assessment was 0.46% (95% CI 0.45–0.48%) in Norway and 3.96% (95% CI 3.91–4.02%) in Spain. The proportion having additional mammographic imaging was 3.46% (95% CI 3.41–3.50%) in Denmark, 4.96% (95% CI 4.90–5.02%) in Norway, and 3.0% (95% CI 2.96–3.05%) in Spain (Table 2).

### Performance of the recall assessment

Positive predictive values of the assessment pathways reflect the overall PPV-1 and PPV-2, affected by the recall rates and higher predictive value in the pathways including biopsies. The IC_pos_ rate was 2.00 after no follow-up in Denmark, 1.66 in Norway, and 0.33 in Spain. The group with short-term follow-up, i.e. 6–12 months, had an IC_pos_ rate of 2.67 (Table 3).

**Table 3:** Screen-detected (SDC) and interval cancers (IC) detected in each diagnostic pathway during two years from the screening in Denmark, Norway and Spain

	Screen-detected cancers/number of recall examinations (PPV-1)			Interval cancers/number of positive screenings not diagnosed with cancer (ICR_pos_)		
	Denmark *N* (%)	Norway *N* (%)	Spain *N* (%)	Denmark *N* (%)	Norway *N* (%)	Spain *N* (%)
No follow-up performed within 6 months or women lost to follow-up	0	7/309 (2.3)	< 5/3016	< 5/248 (~ 2.02)	< 5/302 (~ 1.66)	10 / > 3011 (~ 0.33)
Additional imaging^, ultrasound and biopsy	6125/10,446 (58.6)	4842/11,273 (43.0)	779/1603 (48.6)	43/4321 (1.00)	31/6431 (0.48)	< 5/824 (0.61)
Additional imaging^ and ultrasound	8/12,851 (0.06)	16 /14,629 (0.11)	18/4339 (0.41)	66/12,843 (0.51)	55/14,613 (0.38)	19/4321 (0.44)
Additional imaging^ alone	< 5/671 (< 0.75)	–	8/7726 (0.10)	< 5 / > 884(~ 0.57)	–	27/7718 (0.35)
Ultrasound alone		< 5/2423 (< 0.21)	45 /19,093 (0.24)		7 / < 2418 (~ 0.29)	55/19,048 (0.29)
Ultrasound and biopsy	112/276 (40.6)	325/1416 (23.0)	1305/3731 (35.0)		< 5/1091 (~ 0.46)	13/2426 (0.54)
Additional imaging^ and biopsy	51/66 (77.2)	–	262/821 (31.9)		–	< 5/559 (~ 0.89)
Biopsy alone	48/87 (55.2)	–	480/1129 (42.5)		–	9/649 (1.39)
Early follow-up			51/351 (14.5)			8/300 (2.67)

Due to GDPR, cells with numbers below 5 and the corresponding denominators (regardless of which table they occur in) are masked with < / >

^^^Includes, extra mammography, breast MRIs or tomosynthesis

The sensitivity of the diagnostic follow-up was 99.3–99.4% in all three countries when all three diagnostic elements were included in the follow-up after positive screening (Table 4). In Denmark and Norway, the pathways not including biopsies, i.e. mammography and ultrasound, had the lowest sensitivities of 22.5% and 10.1%, while in Spain pathways including only additional mammography had the lowest sensitivity of 27.0%. The widespread use of only ultrasound in the diagnostic work-up in Spain presented a sensitivity of 45.0%, thus almost similar to the sensitivity of also including additional mammography (48.6%).

**Table 4:** Sensitivity of the recall examination by a diagnostic pathway in Denmark, Norway and Spain (excluding no follow-up)

	Interval cancers and sensitivity among women with a positive screening mammogram (Sens-2)					
	Denmark		Norway		Spain	
	Total number of SDC + IC	Interval cancers *N* (Sens-2)	Total number of SDC + IC	Interval cancers *N* (Sens-2)	Total number of SDC + IC	Interval cancers *N* (Sens-2)
Additional imaging^, ultrasound and biopsy	6168	43 (99.3)	4873	31 (99.4)	< 784	< 5 (~ 99.4)
Additional imaging^ and ultrasound	74	66 (10.1)	71	55 (22.5)	37	19 (48.6)
Additional imaging^ alone	216	< 5 (~ 98%)	–	–	35	27 (27.0)
Ultrasound alone			< 12	7 (~ 41.7)	100	55 (45.0)
Ultrasound and biopsy			< 330	< 5 (~ 98.5)	1318	13 (99.0)
Additional imaging^ and biopsy			–	–	< 267	< 5 (98.1)
Biopsy alone			–	–	489	9 (98.2)
Early follow-up	–	–	–	–	59	8 (86.4)
Total	6458	> 109 (~ 98.3)	> 5286	> 93 (98.2)	> 3089	> 141 (~ 95.4)
Total of pathways including at least one biopsy	> 6341	> 43 (99.2)	5203	36 (99.3)	> 2858	32 (98.8)

Due to GDPR, cells with numbers below 5 and the corresponding denominators (regardless of which tables they occur in) are masked with < / >

^^^Includes, extra mammography, breast MRIs or tomosynthesis

## Discussion

### Main results

We found variations in which assessment procedures were performed after abnormal screening mammography in Denmark, Norway, and Spain. Different recall rates could reflect the choice of diagnostic procedures; in Spain, almost 4% of the screened population underwent an ultrasound examination as the only assessment, which is not recommended in Denmark and Norway. Further assessment was performed within 2 months after screening for most of the cases, in all three countries.

The SDC rates were similar in Denmark and Norway, while the Spanish rate was lower. The IC rate among false positive recalls in Spain was lower than in Denmark and Norway, however, taking the different recall rates into account the sensitivity of the recall assessment seemed higher in Denmark and Norway compared to Spain.

### Comparison with other studies

Results from other population-based screening programmes in Europe reveal very high follow-up rates of 98–99% after recall in line with our findings [23–25]. The high proportion of loss to follow-up in the Spanish population may be due to missing data as the use of private clinics and insurance is more common.

Denmark had the highest proportion of follow-up performed within 30 days and the least difference between false positives and cancer cases in terms of follow-up time. This might be explained by the ‘Cancer Care Pathways’ which were implemented in 2008 in Denmark and the health care institutions undergo strict monitoring regularly secure appropriate and timely diagnostic processes [9]. However, a similar pathway was implemented in Norway in 2015 [26].

It is well known that there may be differences in time to follow-up between women who end up being false positive and those who end up with a cancer diagnosis and the observed differences in our study are in line with other findings [3, 27]. No studies have previously compared follow-up strategies in these countries and assessed differences in cancer detection rates based on different follow-up strategies.

### Clinical implications

SDC rates in Denmark and Norway are comparable even though Norway refers a higher percentage. The background incidence seems to be lower in Norway [28]; thus, it may be concluded that the higher recall rate does lead to more cancers detected, which could indicate that increasing the number of recalls in Denmark could increase the SDC rate and decrease the IC rate. However, the number of recalls needed to detect one cancer should be balanced, and thus, reducing numbers of recalls in Norway could be considered as well.

In Spain, we found a lower rate of SDC compared to Denmark and Norway. Previous data on cancer incidence suggest that the background incidence rate is inferior in Spain compared to Denmark and Norway [29], which could partially explain this difference. In addition, the different IC rates, both IC_rate_ and IC_pos_ were lower in Spain than in Denmark and Norway. Despite the overall sensitivity (Sens-1) in Spain being similar to or even higher than in Denmark, the sensitivity of the recall assessment (Sens-2) was not as high in Spain, partially due to the aforementioned lower detection rate (Table 4). Also, the inferior Sens-2 could be explained by the fact that in Spain some women may leave the screening path after a recall for further assessments and continue follow-up in private clinics. This would inevitably lead to some cancers being diagnosed outside the screening programme, and thereby as IC’s, even though its diagnosis is a direct consequence of a screening recall.

In all three countries, the Sens-2 is low if no biopsies were performed during the diagnostic follow-up given the fact that the total number of cancers diagnosed in these groups are few. The widespread use of assessment only including additional imaging in Spain will inevitably affect the proportion of IC’s and excluding these pathways, led to almost similar Sens-2 in the three countries (Table 4). Also, it should be mentioned that in Spain most screening centres are managed and located within a clinical setting, which may result in the more frequent use of additional imaging procedures. This finding could indicate a potential overuse of non-invasive procedures in their assessment path.

Cancers that occurred after false-positive screening results may be fast-developing cancers or missed cancers at the diagnostic follow-up. Thus, the missed cancers would be the ones susceptible to improved diagnostic assessment. However, we do not know the proportion of missed cancers among the cancers that occurred as interval cancers in this population of women with false-positive screening results. Highly inconsistent estimations of missed cancers in other studies of 25–75% make it difficult to decide the number of women potentially susceptible to improved recall assessment [30, 31].

### Strengths and limitations

This study builds upon high-quality register data in all three countries. To be able to compare results between the countries, careful considerations and attempts to align the inclusion and exclusion criteria have been made. However, we did have some limitations as data availability and definitions differed between the countries. Firstly, we used data from 2016 to 2019 from Denmark and Norway, while the most recent available data from the Spanish population were from 2012 to 2015. This might include some differences in screening methods, referral rates, and diagnostic work-up strategies. Fine needle biopsies were used to a much larger extent in the diagnostic follow-up previously. Secondly, register data from private providers might be less complete. In Denmark and Norway, private providers are rarely used as part of recall assessment after the positive screening, and thus, the problem with missing elements is considered extremely low. In contrast, the high proportion of lost to follow-up in the Spanish population could partly be explained by diagnostic work-up using private insurance which is not always reported to the register. Finally, defining interval cancers based on time to diagnosis in the Danish and Norwegian populations may cause some minor misclassification, since SDC’s detected > 6 months after screening can be falsely categorized as IC’s. However, almost all women have their first follow-up assessment within 3 months, wherefore only very few would have their final diagnosis delayed beyond 6 months.

## Conclusion

This retrospective comparison of diagnostic follow-up performed in three European countries with population-based mammography screening programmes showed large variation in the recall assessment procedures used. Furthermore, positive predictive values, cancer detection rates, and sensitivity differed between the countries and the reasons for that should be studied further. It cannot be ruled out that different recall rates and follow-up strategies affect these performance measures.

## Data Availability

Denmark: The data that support the findings of this study are made available from The Danish Health Data Authority. Restrictions apply to the availability of these data, which were used under license for this study. Data may be available upon reasonable request to The Danish Health Data Authority. Norway: Data from the Cancer Registry of Norway (CRN) have been used in this publication. The interpretation and reporting of these data are the sole responsibility of the authors, and no endorsement by CRN is intended nor should be inferred. Research data used in the analyses can be made available on request to https://helsedata.no/, given legal basis in Articles 6 and 9 of the GDPR and that the processing is in accordance with Article 5 of the GDPR. Spain: Research data used in the analyses can be made available on reasonable request.

## Appendix

See Table

**Table 5:** Diagnostic follow-up pathways during two years from screening and proportions out of women referred for recall assessment within the study period in Denmark, Norway and Spain

		Denmark (*N* = 24,645 women) *N* (%)	Norway (*N* = 30,050 women) *N* (%)	Spain (*N* = 41,809 women) *N* (%)
1	No follow-up in 6 months/lost to follow-up	248 (1.0)	309 (1.0)	3016 (7.2)
2	Additional imaging^, ultrasound and biopsies	10,446 (42.5)	11,273 (37.5)	1603 (3.8)
3	Additional imaging^ and ultrasound	12,851 (52.1)	14,629 (48.7)	4339 (10.4)
4	Additional imaging^ alone	47 (0.2)	–	7726 (18.5)
5	Ultrasound alone	624 (2.5)	2423 (8.1)	19,093 (45.7)
6	Ultrasound and biopsy	276 (1.1)	1416 (4.7)	3731 (8.9)
7	Additional imaging^ and biopsy	66 (0.3)	–	821 (2.0)
8	Biopsy only (i.e. excisional biopsies, stereotactic biopsies)	87 (0.4)	–	1129 (2.7)
9	Early follow-up recommendation*	–	–	351 (0.8)

^Includes, extra mammography, breast MRIs or tomosynthesis

*Only in Spain

5.

## Abbreviations

SDC: Screen-detected cancer
IC: Interval cancer
BI-RADS: Breast Imaging Reporting and Data System
PPV-1: Positive predictive value of the diagnostic follow-up
PPV-2: Positive predictive value of the diagnostic follow-up including at least one biopsy
Sens-1: Sensitivity of screening
Sens-2: Sensitivity of the diagnostic follow-up
MRI: Magnetic resonance imaging
IC_rate_: Interval cancer rate among all negative screening examinations
ICR_pos_: Interval cancer rate among false positive screening examinations
GDPR: General Data Protection Regulation
